# Evolution of the B3 DNA Binding Superfamily: New Insights into REM Family Gene Diversification

**DOI:** 10.1371/journal.pone.0005791

**Published:** 2009-06-08

**Authors:** Elisson A. C. Romanel, Carlos G. Schrago, Rafael M. Couñago, Claudia A. M. Russo, Márcio Alves-Ferreira

**Affiliations:** 1 Department of Genetics, Federal University of Rio de Janeiro, Rio de Janeiro, Brazil; 2 Department of Biochemistry, University of Otago, Dunedin, New Zealand; University of Umeå, Sweden

## Abstract

**Background:**

The B3 DNA binding domain includes five families: auxin response factor (ARF), abscisic acid-insensitive3 (ABI3), high level expression of sugar inducible (HSI), related to ABI3/VP1 (RAV) and reproductive meristem (REM). The release of the complete genomes of the angiosperm eudicots *Arabidopsis thaliana* and *Populus trichocarpa*, the monocot *Orysa sativa*, the bryophyte *Physcomitrella patens*,the green algae *Chlamydomonas reinhardtii* and *Volvox carteri* and the red algae *Cyanidioschyzon melorae* provided an exceptional opportunity to study the evolution of this superfamily.

**Methodology:**

In order to better understand the origin and the diversification of B3 domains in plants, we combined comparative phylogenetic analysis with exon/intron structure and duplication events. In addition, we investigated the conservation and divergence of the B3 domain during the origin and evolution of each family.

**Conclusions:**

Our data indicate that showed that the B3 containing genes have undergone extensive duplication events, and that the REM family B3 domain has a highly diverged DNA binding. Our results also indicate that the founding member of the B3 gene family is likely to be similar to the ABI3/HSI genes found in *C. reinhardtii* and *V. carteri*. Among the B3 families, ABI3, HSI, RAV and ARF are most structurally conserved, whereas the REM family has experienced a rapid divergence. These results are discussed in light of their functional and evolutionary roles in plant development.

## Introduction

The B3 domain was first identified in the *VIVIPAROUS* (*VP1*) gene from *Zea mays*, which contains three basic regions designated as B1, B2 and B3 [1] and the *VP1* orthologue *ABI3* (*ABSCISIC ACID*-*INSENSITIVE3*) from *Arabidopsis thaliana* [2]. Five major classes of genes containing the B3 domain have been identified to date based on their similarities and domain structures. These include proteins from the ABI3/VP1 [1], HSI (High-level expression of sugar-inducible gene) [3], [4], RAV (Related to ABI3/VP1) [5], ARF (Auxin Response Factor) [6] and REM (Reproductive Meristem) [7] families. B3 DNA binding specificity has been studied in three families: ABI3, RAV and ARF. The B3 domain of the ABI3 family recognizes the Sph/RY element of the CATGCA sequence [1], [8], [9], [10]. Proteins of the RAV family are characterized by the presence of an N-terminal DNA binding AP2/EREBP domain that recognizes the CAACA sequence and a C-terminal B3 domain that recognizes the CACCTG sequence [5]. The ARF family is characterized by the presence of an N-terminal B3 domain that recognizes the TGTCTC sequence (auxin response elements - AuxREs), a middle domain that is highly divergent and works as a transcriptional activation or repression domain [11], and a C-terminal dimerization domain containing motifs III and IV similar to motifs of Aux/IAA proteins [6].

Interestingly, it has been shown that B3 domains from distinct families bind to different DNA sites. Yet, these proteins share a common structural framework for DNA-recognition. Analysis, by NMR spectroscopy, of the structure of the B3 domain of the At1g16640 protein from Arabidopsis [12], a member of the REM family, revealed that it has the same novel fold as RAV1 with seven-stranded β-sheet arranged in an open barrel and two short α-helices. Nevertheless, this particular gene (At1g16640) has a remarkably distinct amino acid sequence from others in the superfamily. This has raised doubts to whether this domain has the ability to bind to DNA. However, it has been showed that VRN1 (VERNALIZATION1), a member of the REM family, binds DNA *in vitro* in a non-sequence-specific manner [13], which indicates that perhaps specific DNA binding has been lost, while retaining general DNA binding.

Proteins with the B3 domain are involved in many plant processes. Three transcriptional activators *FUSCA3* (*FUS3*), *LEAFY COTYLEDON2* (*LEC2*) and *ABSCISIC ACID INSENSITIVE3* (*ABI3*) and three repressors *HIGH-LEVEL EXPRESSION OF SUGAR-INDUCIBLE GENE 2* (*HSI*), *HSI L1* and *HSIL2* or *VP1*/*ABI3*-*LIKE* (*VAL*) from the ABI3 and HSI/VAL families were shown to be involved in seed development and maturation [3], [4], [14], [15], [16]. RAV genes are not well characterized, but some of them have been showed to be involved with growth, development and flowering time [17], [18], [19]. The best studied family of B3 is the ARF family, which regulates a range of responses to auxin and have additional systems of regulation [20], [21], [22], [23]. On the other hand, the major REM family has no functional information available up to now [7], except to the *VRN1* (*VERNALIZATION 1*) which acts promoting flowering [13], [24]. The B3 proteins functionally characterized from the ABI3, HSI, RAV and ARF families have shown that they are mainly involved in hormone, signaling pathways such as those for auxin, abscisic acid, brassinosteroid and gibberellin.

In this work, we explored the evolution of these important proteins in eight plant species for which the genome has been completely sequenced, ranging from the green algae *Chlamydomonas reinhardtii* to the eudicot *Arabidopsis thaliana*. Our analyses help to elucidate the origin and the diversification of the B3 superfamily. Additionally, we studied the conservation of the B3 domain during evolution, and integrated the analyses of B3 evolution with the well-characterized families AP2/EREBP (APETALA 2/ethylene responsive element binding protein) and ARF. The phylogenetic relationships between the B3 members are discussed in the context of the functional diversity among the genes.

## Results

### Identification and classification of the B3 superfamily in Arabidopsis, poplar, rice, moss and algae

**Figure 1:**
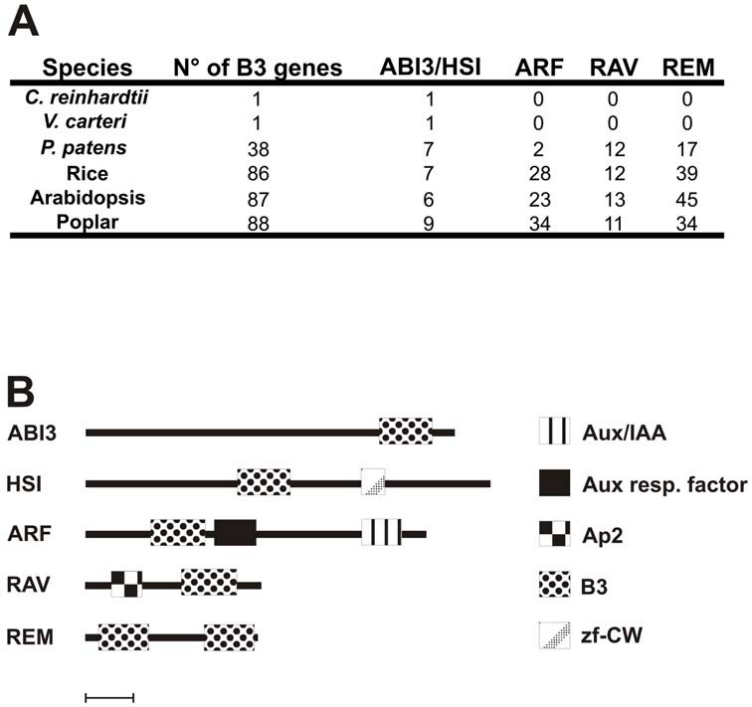
Table with total number of B3 protein per species and diagram of domain organization of the B3 family based on their protein sequences. A – The identification of all proteins containing the B3 domain was conducted using the INTERPRO code of the B3 in respective species web site. Complete information of the genes and domains are in tables S1, S2, S3 and S4. B – Diagram of the domains organization of selected B3 proteins of each family. All selected proteins are from Arabidopsis, contain the typical domains found in each respective family and have been studied before: ABI3, At3g24650 (ABI3); HSI, At2g30470 (HSI2/VAL); ARF, At1g59750 (ARF1); RAV, At1g13260 (RAV1); REM, At3g18990 (VRN1). Proteins of REM family have one to 11 B3 domains. The domains are shown in scale and solid lines denote protein sequences. Bar – 100 amino acids.

Identification of all the proteins containing the B3 domain was conducted using the INTERPRO code of the B3 domain in the respective species website (see material and methods). A summary of the results is shown in figure 1, as well as a diagram of the protein domain organization of all B3 families. The identification of B3, encoding genes was based on the genome annotation (see material and methods), and checked by the use of the Pfam and INTERPRO programs (see material and methods). All identified genes were analysed for the presence of the B3 domain using PFAM program and the significant E-value of B3 domain is showed for all genes (Table S1, S2, S3, S4). The gene models for all species selected for this work were consolidated and annotated using information of EST assemblies and full-length cDNAs, which ensures that the genes are expressed [25], [26], [27], [28], [29].

In the *P. patens* genome, we found 38 B3 genes [25]. Comparative analysis with other plant genomes also revealed that this species possesses the typical families found in angiosperms (see below), but with a lower number of members: ABI3 (5 loci); HSI (2 loci); RAV (2 loci); ARF (12 loci) and REM (17 loci). As this bryophyte has the lowest number of REM genes among all multicellular species with available genomic information, it was considered the basal species in this study. An initial comparison of all REM proteins of *P. patens*, Arabidopsis, poplar and rice identified four distinct REM classes of B3 proteins shared among these species (Figure S1A). *P. patens* possesses two classes, REM I and III, that are found in other species studied here (see below). On the other hand, the *P. patens* genome has two specific classes not found in the other species studied, named REM II (7 loci) and REM IV (3 loci) (Figure S1 and Table S1).

Using the TAIR annotation consortium, it was possible to identify 87 B3 proteins in the Arabidopsis genome [29]. Table S2 shows these Arabidopsis proteins classified into the five known families: ABI3 (3 loci), HSI (3 loci), RAV (13 loci), ARF (23 loci), REM (45 loci). Among the B3 families, the REM family is the most numerous and divergent, not only in Arabidopsis, but in all species included in this work. A recent characterization of the B3 family in plants found 28 additional genes with similarity to the B3 superfamily in Arabidopsis [30]. However, these genes do not have the typical B3 domain and they were not included in our analysis (Table S5).

In order to illuminate the evolution of the REM family, we performed the phylogenetic analysis with both complete sequences of REM proteins, and also with the B3 domains present in each protein. As several members of the REM family have more than one B3 domain, we decided to treat them as distinct operational taxonomic units (OTUs). This strategy allowed us to better identify the REM classes among the different species. The first step of our phylogenetic analysis was the comparison of the protein sequences of rice, poplar and Arabidopsis with *P. patens* protein sequences (data not shown). After this initial analysis, which allowed us to identify common groups among bryophytes and flowering plants, we performed a broader evolutionary comparative analysis including all species for each REM class (data not shown). This study revealed that REM class I is the only group in common among *Arabidopsis thaliana*, *O. sativa*, *P. trichocarpa* and *P. patens* species (see below). These studies were supported by the number of B3 domains in each protein, alignments and bootstrap analyses in phylogenetic trees constructed using the neighbor-joining method [31] and *p*-distance on the Mega 4 program [32]. The topological stability was confirmed by phylogenetic analyses using the JTT model [33].

Phylogenetic analysis of the REM family in Arabidopsis revealed the existence of seven distinct REM classes (Figure S1B) (REM I and REM V to REM X); some of these classes are also found in rice and poplar. REM VII (8 loci), REM IX (15 loci) and REM X (2 loci) are found exclusively in Arabidopsis. REM genes have been previously identified and classified in a previous work [7]; however, our analyses, reveal the need for renaming the genes to assure a more meaningful classification.

Our analysis of the poplar genome [26] revealed 88 B3 genes belonging to the same five typical families of Arabidopsis: ABI3 (2 loci), HSI (7 loci), RAV (11 loci), ARF (34 loci) and REM genes (34 loci) (Table S3). As in Arabidopsis, PtREM-class proteins are characterized by genes with multiple B3 domains. Arabidopsis and poplar have the REM class VI proteins in common. We also have identified a new poplar-specific REM class with eight genes, named REM XI.

We identified 86 genes of the B3 superfamily in the rice genome [27], [28]: ABI3 (5 loci), HSI (2 loci), ARF (28 loci), RAV (12 loci) and REM (39 loci) (Table S4). Our analysis reveal that rice and moss share REM III class and it possesses two species-specific REM classes, named REM XII (2 loci) and XIII (15 loci). Swaminathan and collaborators [30] found 15 RAV loci, three of them were excluded from our analysis due to the lack of the typical B3 domain (Table S5).

In order to address B3 superfamily evolution, we searched for B3 genes in the genomes of *C. reinhardtii*, three green algae species (*Volvox carteri*, *Ostreococcus tauri*, *Ostreococcus lucimarinus)* and one red algae, *Cyanidioschyzon merolae* (JGI; [34], [35]). Among these genomes, we were able to find single B3 representatives in *C. reinhardtii* and *V. carteri* genomes (JGI databases). Comparative analysis of these algal B3 genes with all the B3 genes of the other species indicates that those two genes are more similar to the HSI and the ABI3 families of land plants (see below).

### Gene structure evolution of B3 domain ORFs

**Figure 2:**
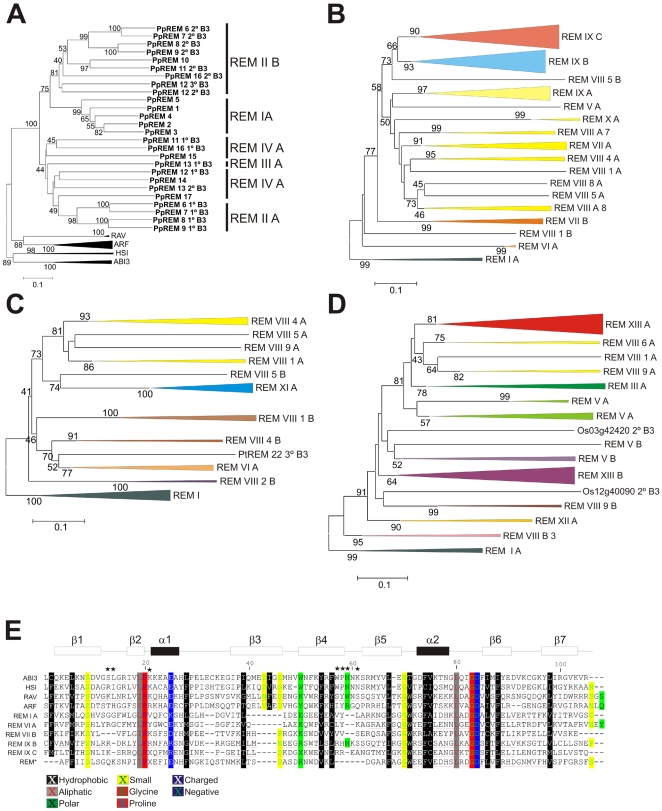
Phylogeny of B3 domains sequences. A, Rooted in ABI3/HSI Neighbor-joining tree of all five B3 families based on the B3 domain amino acid sequences of moss. B, Rooted in REM I class Neighbor-joining tree of all B3 domains members of REM family from Arabidopsis. C, Rooted in REM I class Neighbor-joining tree of all B3 domains member of REM family from poplar. D, Rooted in REM I class Neighbor-joining tree of all B3 domain member of REM family from rice. A, B, C and D - bootstrap values from 1,000 replicates. The scale bar represents a 0.1 estimated aminoacid substitution per residue. Bootstrap values >40 are shown. The color chosen for each group of homologous is used to indicate the B3 domain types in figures 3 and 4. E, Alignment of the COBBLER-derived B3 domain sequences from *A. thaliana* proteins. REM* represent an alignment with all B3 domain from REM X A, REM VIII A and REM VII A types. All of them have one common point with 50% of boostrap in Figure 2B. The black bar and white bars represent predicted α-helix and β-sheet regions, respectively, within the B3 domain [37]. The red line underneath of sequences indicates residues of the embedded COBBLER consensus block. Black Stars represent amino acid residues that make direct contact with DNA in the RAV protein [37]. Meaningful similarities are indicated by color bars.

In addition to the classification in distinct classes, we have used letters to classify each B3 domain according to the position in the protein, i.e., A indicates the closest B3 domain to the protein's N-terminal region. B3 domains of proteins with only one domain were also named A. Phylogenetic analysis of the B3 domains of all five B3 families of *P. patens* showed well supported family groups and specific REM B3 domains (Figure 2A). This result suggests that the REM family underwent extensive duplication events before the appearance of moss. Our analysis revealed the existence of five different types of B3 domains in moss (REM I; REM IIA; REMIIB; REM III A; REM IV A). It is interesting to note that some PpREM genes belonging to different REM classes have similar B3 domains such as the 1^st^ B3 domain of PpREM16 (REM IV) and the 1^st^ B3 domain PpREM11 (REM II) that are grouped together in REM IV A.

Evaluation of the REM family in Arabidopsis, poplar and rice revealed that the number of members of this family increased dramatically when compared with moss. Besides the elevated number of members, REM family members are also highly divergent. These two characteristics hampered the phylogenetic analysis of this family. Only after extensive analysis of all B3 domains of the species studied in this work and species to species comparisons, was it possible to achieve a robust classification. As resulted, we classified all B3 domains of *A. thaliana* (Figure 2B; Figure S1C), *P. trichocarpa* (Figure 2C) and *O. sativa* (Figure 2D; Figure S1D).

**Figure 3:**
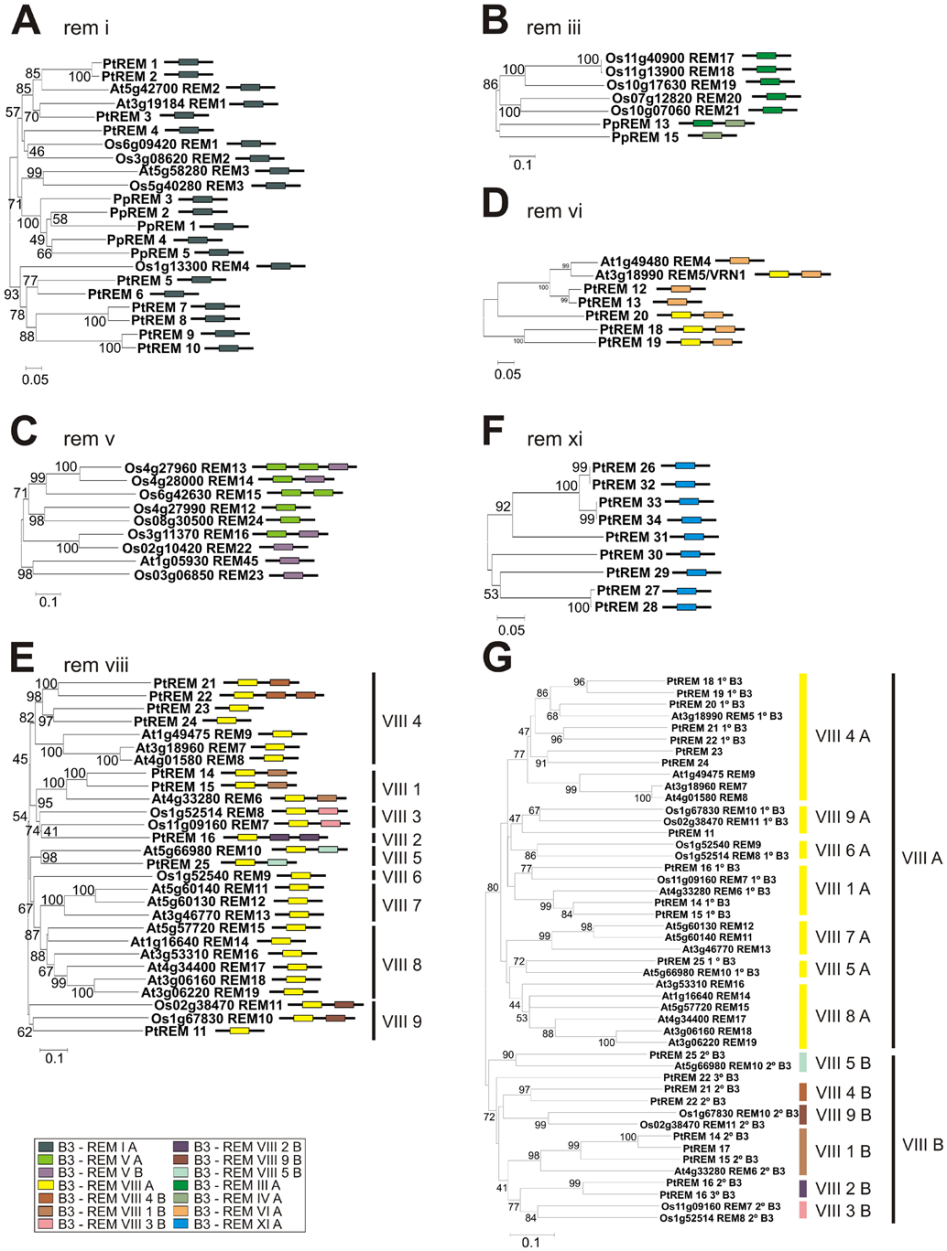
Phylogenetic relationships among Arabidopsis, poplar, rice and *P*. *patens* B3 protein sequences of the REM family. A, Unrooted Neighbor-joining consensus tree of the entire amino acid sequences of Arabidopsis, poplar, rice and *P. patens* REM I class proteins. B, Unrooted Neighbor-joining consensus tree of the entire amino acid sequences of rice and *P. patens* REM III class proteins. C, Unrooted Neighbor-joining consensus tree of the entire amino acid sequences of Arabidopsis and rice REM V class proteins. D, Unrooted Neighbor-joining consensus tree of the entire amino acid sequences of Arabidopsis and poplar REM VI class proteins. E, Unrooted Neighbor-joining consensus tree of the entire amino acid sequences of Arabidopsis, poplar and rice REM VIII class proteins divided in specific groups. PtREM17 was excluded from this analysis because the sequence is short and do not makes any common alignment. F, Unrooted Neighbor-joining consensus tree of the entire amino acid sequences of poplar REM XI class proteins. G, Unrooted Neighbor-joining tree of all B3 domains members of REM VIII class from Arabidopsis, poplar and rice showing two groups well supported (REM VIII A and REM VIII B). Additionally, REM VIII B was classified in other specific groups as supported in Figure 3E and other analysis (see text). The number and type of B3 domain classified in Figure 2 is represented for each gene with different colours. Bootstrap values from 1,000 replicates were used to assess the robustness of the trees. Bootstrap values >40 are shown. The scale bar represents a 0.05 estimated amino acid substitution per residue.

The phylogenetic analysis of the REM B3 domain sequences of *A. thaliana* (Figure 2B) indicated that five distinct types of B3 domains are well supported: REM I A, VI A, VII B, IX B and IX C. In addition, several other groups with a high similarity among them are observed: REM V A, X A, IX A, VII A, and VIII A. In spite of this high divergence among several B3 domain types, the groups and the tree topology is well supported by comparison between species, such as Arabidopsis and poplar (Figure 3G). As described above, all B3 domains of the AtREM I class belong to the REM I A type and share homology with moss and other species (Figure 3A). In the REM VI class genes, all B3 domains belong to the type REM VI A, excluding the first domain of *VRN1* (*REM 5*) and the PtREM proteins 18 to 20, which have an additional type REM VIII 4 A B3 domain in their amino terminal regions (Figure 3D and G). The vast majority of the Arabidopsis REM VIII class members have only one B3 domain. On the other hand, most of the rice and poplar genes possess two B3 domains (Figure 3E). Our broad comparative phylogenetic analysis with B3 domains from Arabidopsis, rice and poplar (Figure 3G, 2B, 2C, 2D) shows that the type REM VIII B3 domains can be grouped in sub-types. These sub-types are also well supported by our phylogenetic analysis (Figure 3G). The distinct REM VIII domain sub-types were identified by Arabic numerals after the Roman numeral classification of the B3 types. The nomenclature presented reflects the complexity due to recent divergence of the class VIII REM genes. *AtREM6*/*PtREM14*/*PtREM15* and *AtREM10*/*PtREM25* possess an additional C-terminal B3 domain conserved between Arabidopsis and poplar (Figure 3E). These similarities indicate the existence of these genes before the divergence of Arabidopsis and Poplar.

**Figure 4:**
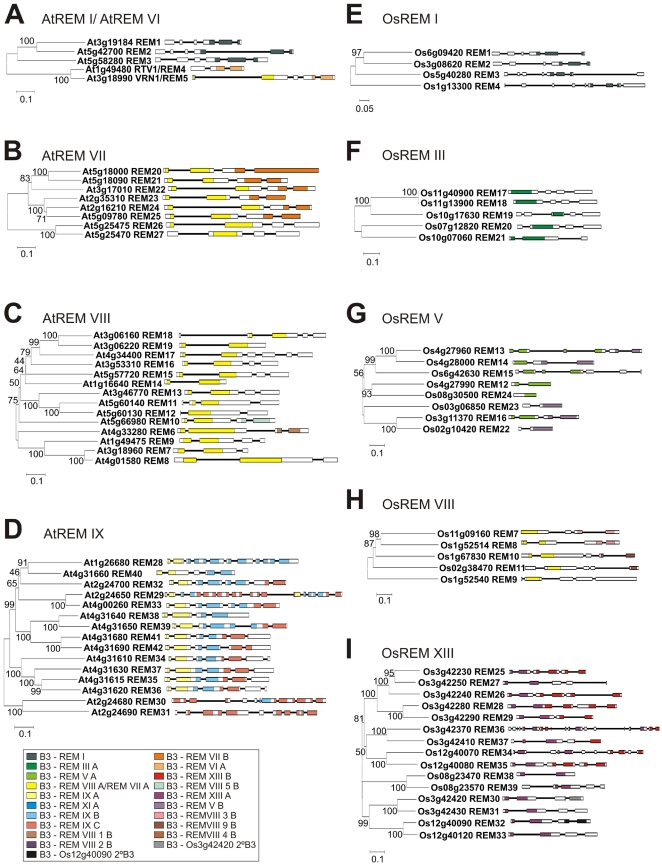
Phylogenetic relationships and exon/intron structure with B3 domain localization in each gene for REM family of Arabidopsis and rice. A, Unrooted Neighbor-joining tree of the entire amino acid sequences of Arabidopsis REM I and REM VI proteins. B, Unrooted Neighbor-joining tree of the entire amino acid sequences of Arabidopsis REM VII proteins. C, Unrooted Neighbor-joining tree of the entire amino acid sequences of Arabidopsis REM VIII proteins. D, Unrooted Neighbor-joining tree of entire amino acid sequences of Arabidopsis REM IX proteins. E, Unrooted Neighbor-joining tree of the entire amino acid sequences of rice REM I proteins. F, Unrooted Neighbor-joining tree of the entire amino acid sequences of rice REM III proteins. G, Unrooted Neighbor-joining tree of the entire amino acid sequences of rice REM V proteins. H, Unrooted Neighbor-joining tree of entire amino acid sequences of rice REM VIII proteins. I, Unrooted Neighbor-joining tree of entire amino acid sequences of rice REM XIII proteins. Bootstrap values from 1,000 replicates were used to assess the robustness of the trees. Bootstrap values >40 are shown. The scale bar represents a 0.1 estimated amino acid substitution per residue. The phylogenetic tree and exon/intron structure with domain localization of each B3 domain type are shown. Each colored box represent one B3 type domain, as indicate in the figure. The different colours of REM B3 domains per protein were based on different class found in phylogenetic analysis of REM B3 domains showed in Figure 2B, 2C, 2D. MIPS *Arabidopsis thaliana* and *Orysa sativa* database was used for exon/intron structure information [87]. As it is not possible to construct phylogenetic tree with less than 4 genes, specific classes with few members of classes were not showed in this figure such as AtREM X and OsREM XII.

The AtREM VII and AtREM IX classes are exclusively found in Arabidopsis and most of them possess more than one type of B3 domains in the same protein (Figure 4B and D). AtREM VII genes have the B3 domain in amino terminal position (REM VII A) very similar to REM VIII A type (for sake of simplicity the same colour was attributed for both domains types in Figures 2B and 4B). The type REM VII B domain probably appeared later, given that the class VII and VIII genes possess similar domains in their N-terminal region (Figure 2B, Figure S1B). Three types of B3 domains are exclusively found in the AtREM IX class: REM IX A, REM IX B and REM IX C (Figure 2B and 4D). Analysis of the *AtREM41* and *AtREM42* genes revealed that the 1^st^, 2^nd^ and 3^rd^ B3 domains are much more closely related to each other than to other B3 domains, indicating a recent gene duplication event (Figure 4D). *AtREM33* and the C-terminal of *AtREM29* are very similar and may also be the result of a gene duplication event. In addition to complete gene duplication, many REM IX genes have undergone internal B3 domain duplication. This process can be observed in *AtREM28*, *AtREM29* and *AtREM32* (Figure 4D), in which the B3 domains display a higher level of similarity among themselves than to other B3 genes (Figure 4D). The Arabidopsis REM X class has two genes with just one B3 domain which encodes a longer polypeptide than the regular B3 domain: REM 43 (At1g20600) and REM 44 (At4g03170) (Table S2). These B3 domains also possess differences in the amino acid sequences that disturb the alignment and topology of the tree for the REM family.

The phylogenetic analysis of B3 domains in poplar also showed the existence of many distinct B3 domains that are well supported: the REM I A, REM VI A, REM XI A, and REM VIII A types (Figure 2C). The members of the REM XI class possess just one B3 domain and this class is exclusively found in poplar (Figure 3F). As expected, Arabidopsis and poplar share several homologous genes. They are grouped in the classes REM VI and VIII (Figure 3D and 3E).

The phylogenetic analysis of the full-length B3 sequences revealed that rice also presents REM I class genes (Figure 3A). OsREM III genes have just one B3 domain and are similar to members of PpREM III class (Figure 3B). Additionally, the OsREM V genes also share homology with the AtREM V genes (Figure 3C). OsREM XII and OsREM XIII are classes exclusively found in rice (Figure 2D and 4I). The rice B3 domain phylogenetic analysis showed the existence of ten different types of REM B3 domains (Figure 2D). The REM XIII class contains several members and they possesses more than one B3 domain, which can be divided into two different types of B3: REM XIII A and XIII B. There are also two highly divergent REM XIII domains: the 2^nd^ B3 domains of *OsREM32* and *OsREM30* that did not group together (Figure 2D). The analysis of the internal B3 domain duplication events was also conducted for rice (Figure 2D; data not shown). For example, the B3 domains of the following genes are more closely related to each other than to any other B3 domain, indicating duplication events: *OsREM1* and *OsREM2*; *OsREM20* and *OsREM21; OsREM17* and *OsREM18*; 1^st^ B3 domain of *OsREM10* and 1^st^ B3 domain of *OsREM11; OsREM9* and 1^st^ B3 domain of *OsREM8*; the *REM33* and 1^st^ B3 domain of *REM32*; and finally the 2^nd^ B3 domain of *OsREM10* and the 2^nd^ B3 domain of *OsREM11* (Figure 4E to I, data not shown).

To explore the amino acid sequence differences among the B3 domains of ABI3, HSI, RAV, ARF and the distinct REM B3 domain types, we aligned the consensus sequences of the B3 domains of these proteins (Figure 2E). The consensus sequences were generated by the COBBLER (Consensus Biasing By Locally Embedding Residues) program [36]. Among the distinct types of B3 domains, REM I A and REM VI A are the most similar to the B3 domain of RAV, ABI3, HSI and ARF families. This is consistent with by the higher similarity in the residues predicted to bind DNA in the RAV1 protein [37].

### Timing of duplication events

**Figure 5:**
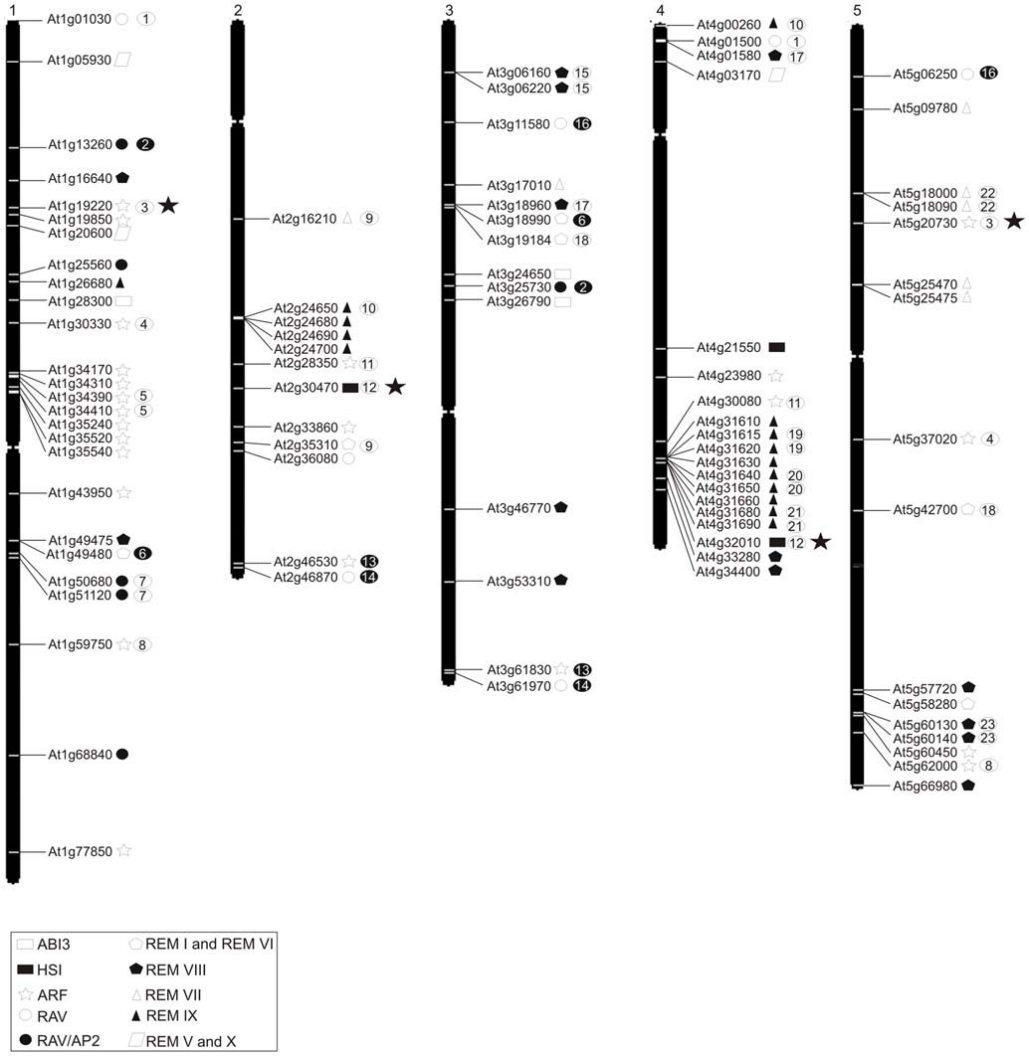
The locations of the B3 superfamily genes on the Arabidopsis chromosomes. The chromosome number is indicated at the top of each chromosome. The chromosomal positions of the B3 genes are indicated by their locus identifier. Families are shown with a box in the corresponding gene (see legends). Duplicated B3 genes identified by our study are designated by numbers in open circles. B3 genes that participated of the most recent duplicated segmental region studied by Blanc et al. [38] are identified by a number in solid black circles. B3 genes that suffered duplication according to our study and that were considered ancient duplicated genes studied by Blanc et al. [38] were marked by a black star.

Analysis of the chromosomal segmentation data [38] (see material and methods) indicates that seven B3 genes (*RAV1*/*RAV1-like*; *VRN1*/*RTV1*; *RAV2*/*RAV2-like*; *AtARF11*/*AtARF18*; *NGA1*/*NGA2*; *RAV-like2*/*RAV-like3*; *AtREM36*/*AtREM29*) were duplicated in a recent genome duplication, around 24–40 million years ago, before the *Arabidopsis*/*Brassica rapa* split (Figure 5). The chromosomal location of the duplication events showed that part of chromosomes 2 and 4 underwent duplication events that resulted in the duplication of REM IX genes, a class of B3 genes exclusive to Arabidopsis (Figure 5). In addition, REM IX genes also underwent, as mentioned before, recent tandem duplications, since they show similar exon/intron structures with conserved number and position of B3 domains (Figure 4D). The number of synonymous substitutions/site/year revealed that class REM IX genes had underwent a duplication event about 4 to 14 million years ago, corroborating the hypotheses mentioned before that specific class of REM genes result from recent duplication events (Table S6). We also detected other recent duplication events in REM members that happened at different times such as: *AtREM8* and *AtREM9*; *AtREM18* and *AtREM19*; *AtREM20* and *AtREM21*; *AtREM23* and *AtREM24* and *AtREM26* and *AtREM27*. The data confirmed that these genes appeared recently in the Arabidopsis genome. Additionally, our analysis uncovered B3 genes from other families that have underwent recent duplication in different chromosomes, as well as old duplication events (Table S6).

The number of synonymous substitutions/site/year was also calculated for other species and we found several B3 duplication events in the rice genome (Table S6). The most important finding is that many genes from REM XIII were duplicated 62 to 18 MYA. These genes are located in chromosomes 3 or 12, and some of them display a high sequence similarity and are also clustered together. These results strongly suggest that these genes originated from tandem duplication events during the whole genome duplication [39] event. A similar analysis in poplar revealed that genes from the REM VI, VIII A 1, VIII A 5 and XI classes appeared between 88 to 33 MYA, indicating that these genes were duplicated before the last whole genome duplication [40]. For moss, we found few recent duplication events in the ARF and REM families dating 49 MYA. These data support our phylogenetic tree comparing all species, and show that several duplication events happened after the monocot-eudicot split and that many of them are species-specific as expected.

### Evolution of B3 domain genes during plant evolution

**Figure 6:**
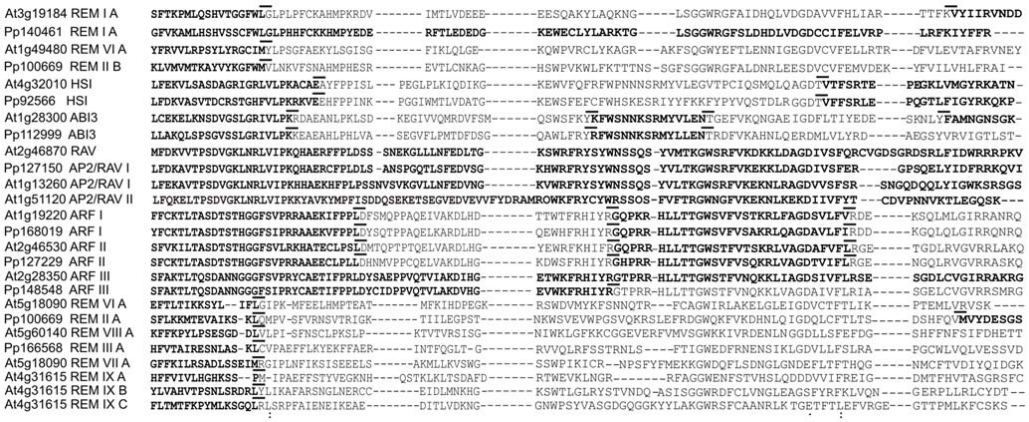
Alignment of amino acid sequences and exon/intron structures of B3 domain from a representative member of all *A. thaliana* and *P. patens* groups. Gaps are indicated by dashes. Underlined letters denote splicing sites. Bold and regular letter were used to help visualization of different exons.

The comparison of splice sites of the families of B3 proteins in *A. thaliana* and *P. patens* genomes revealed gene structure divergence of the B3 domain during plant evolution (Figure 6, Figure 4 and Figure S2). The B3 domain of the REM I class from Arabidopsis is spliced in three exons, whereas the B3 domain of moss is intronless. In the REM II class of moss and REM VI class of Arabidopsis, the B3 domains are spliced in two exons at the same position. For the B3 domains of the REM VII, VIII, IX class of Arabidopsis and REM III of moss, a nearly identical gene structure is observed (two exons). In addition, all RAV family members in Arabidopsis and moss are intronless. The B3 domains of the ARF I and ARF II class of Arabidopsis and moss also have the same splice sites. However, the gene structure of the B3 domain of the Arabidopsis ARF III class contrasts with the moss one: since the former is intronless while the latter is divided in two exons. The splice site is similar to the one observed between the second and third exons in the ARF I and II class. The splice site in the B3 domain of the ABI3 class is nearly identical in Arabidopsis and moss, with only minor differences between the ABI3 and HSI classes (Figure 6).

**Figure 7:**
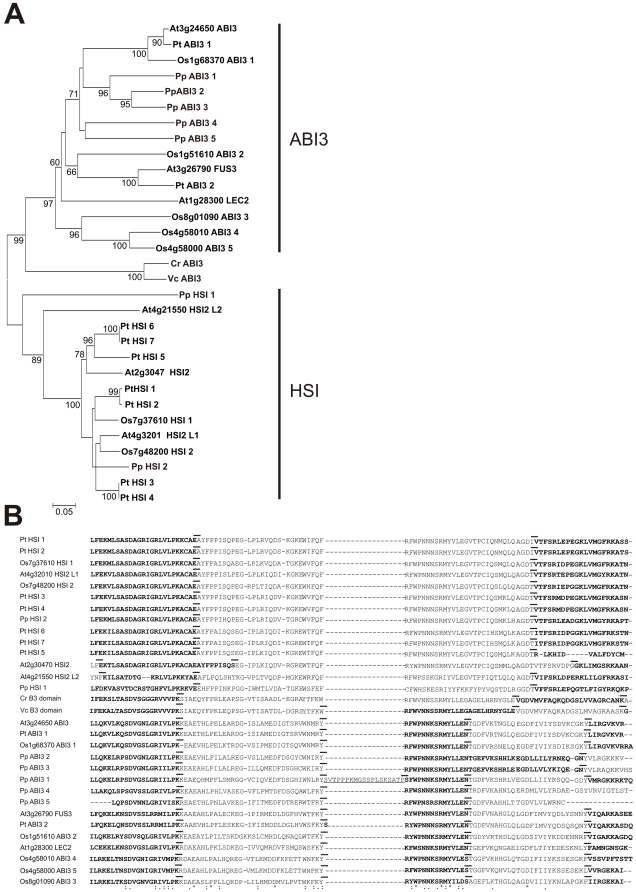
Phylogenetic relationship and exon/intron structure of B3 domains from ABI3 and HSI family in *C. reinhardtii*, *V. carteri*, *P. patens,* rice, poplar and *Arabidopsis*. A, Unrooted Neighbor-joining tree of the B3 domain from *C. reinhardtii*, *V. carteri, P. patens,* rice, poplar and *Arabidopsis*. Bootstrap values from 1,000 replicates were used to assess the robustness of the trees. Bootstrap values >50 are shown. The scale bar represents a 0.05 estimated amino acid substitution per residue. B, Alignment of amino acid sequences and exon/intron structures of B3 domains from all *C. reinhardtii*, *V. carteri*, *P. patens,* rice, poplar and *Arabidopsis* species. Gaps are indicated by dashes. Underlined letters denote splicing sites. Bold and regular letters were used to help visualization of different exons.

The phylogeny and gene structure of B3 domains among *C. reinhardtii*, *V. carteri*, *P. patens*, *O. sativa*, *P. trichocarpa* and *A. thaliana* supports a distinction between ABI3 and HSI gene families (Figure 7A). The ABI3 family has lineage-specific sequences in rice and *P. patens*. The HSI family is present in *Arabidopsis*, poplar, rice and *P. patens* and many of these genes contain one additional domain named zfCW (zinc finger) (Tables S1, S2, S3, S4). The B3 genes found in *C. reinhardtii* and *V. carteri* are spliced in four exons and are more similar to ABI3 and HSI than to any B3 family (Figure 7B). Closer comparative analysis of exon/intron structure among ABI3 family of Arabidopsis, poplar, rice and *P. patens*, and the one B3 gene of *V. carteri* and *C. reinhardtii* showed the same splicing site in the first exon. These results suggest that ABI3 may be the ancestral family of the B3 superfamily. However, when we evaluated the amino acid similarity between the ABI3 and HSI groups and algae proteins, we found out that the ABI3 is more similar to HSI than to the algae proteins (data not shown). This result is consistent with a model in which the putative ancestor B3 gene was similar to *C. reinhardtii* and *V. carteri* B3 genes and it had undergone duplication leading to the formation of the HSI and ABI3 B3 families. HSI family genes in all species have the same splice site position among them, with the exception of *HSI2* and *HSI2 L2* from Arabidopsis, which have minor differences (Figure 7B). The minor differences found in this group may be the result of Intron Sliding (IS) [41].

Comparative phylogenetic analysis of the B3 proteins of the ARF family among different species was performed (Figure S3). Because of the large number of genes in this family, we focused the phylogenetic analysis of the ARF family only on the *Arabidopsis* and *P. patens* genes (Figure S3). Our analysis revealed three classes of ARF genes (I, II and III). In class III, two genes of *P. patens* (*PpARF11* and *PpARF12*) have the B3 domain divided into two exons and belong to the sister group of Arabidopsis *AtARF10* and *AtARF16* that possess an intronless B3 domain. A group of six genes of *P. patens* (*PpARF1*, *PpARF2*, *PpARF3*-6) belongs to the sister group in Arabidopsis (*AtARF6* and *AtARF8*) and a group of four class II genes of *P. patens* (*PpARF7-10)* belong to the sister group of IIB class in Arabidopsis (*AtARF1*, *AtARF2*, *AtARF9*, *AtARF11* and *AtARF18*).

The phylogeny of the RAV family in Arabidopsis, poplar, rice and moss supported two classes named I and II (Figure S4). Class I has AP2 and B3 domains in the same protein of all species, including two genes of *P. patens* (*PpRAV1* and *PpRAV2*). Genes of *P. patens* have the AP2 and B3 domains separated by an intron (data not shown) and, as mentioned before, intronless B3 domains. The class II members display the same domain structure of class I, but also display minor amino acid sequence differences and do not have AP2 domain (Figure 6 and S4).

### Modelling of the B3 domains of families and REM classes

**Figure 8:**
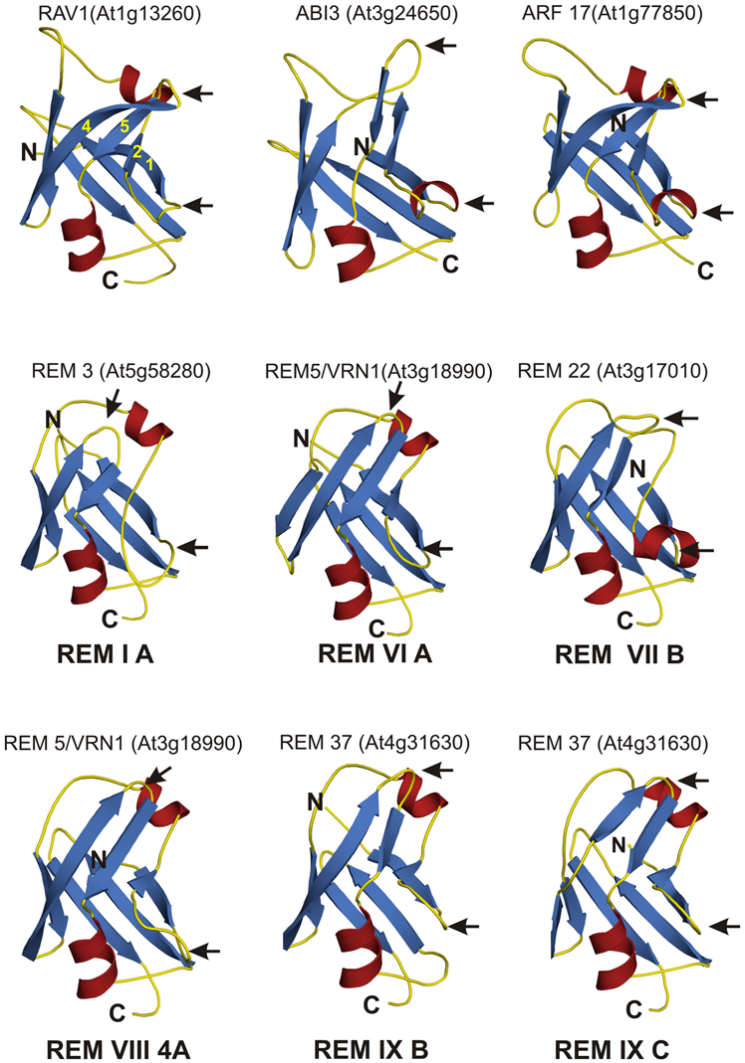
B3 domain modelling of RAV1, ABI3, ARF17 and six distinct REM domain types (REM3, VRN1/REM5, REM22 and REM37). These proteins belong to different B3 families and contain most of the types of B3 domains identified by our phylogenetic analysis. The NMR solution structures for the Arabidopsis protein REM14 (residues 1 to 102; PDB ID 1YEL) or RAV1 (residues 182 to 295; PDB ID 1WID) were used as template. The REM proteins evaluated contain identical structure to RAV1, although they present poor sequence conservation in the two loops (between β strand 1 and 2 and between β strand 4 and 5) that are proposed, in the model, to interact with the DNA (arrows). In addition, the loops are also shorter when compared with member of RAV, ARF, ABI3 and HSI families.

We performed the modelling analysis of B3 domains from different families (ARF and ABI) and for Arabidopsis REM classes (REM I A, REM VI A, REM VII B, REM IX A, REM IX B and REM IX C). B3 domains from selected members were defined as target sequences (see Material and Methods). The NMR solution structures for the Arabidopsis protein REM14 (residues 1 to 102; PDB ID 1YEL) or RAV1 (residues 182 to 295; PDB ID 1WID) were used as template [12], [37]. Despite their low sequence identity, both structures display a common fold; a seven-stranded open beta-barrel and two alpha-helices located at the ends of the barrel [12], [37]. The comparison of the ARF17 B3 and ABI3 domains and the previous determined structure of RAV1 reveal significant structural homology (Figure 8). These three proteins contain identical structure and high sequence conservation in the two loops (between β strand 1 and 2 and between β strand 4 and 5) that are proposed, in the model, to interact with the DNA. As previously shown [37], B3 domains of ARF and ABI3 are very similar to RAV B3 what is coherent with the common structural framework of DNA binding. The structure of At1g16640 (REM14) was shown by Waltner and collaborators [12] to contain a nearly identical structure to RAV1, although the two loops are shorter and present a very limited similarity. REM14 belongs to the REM VIII class of proteins and contain one REM VIII 8 A (Figure 3G). In order to investigate the possible variability of the B3 structure in the REM family, we performed the modelling of seven distinct B3 domains of six different B3 domains of four at REM proteins (REM3, VRN1/REM5, REM 22 and REM37 – Figure 8). These proteins belong to different REM classes and contain all possible types of B3 domains identified by our phylogenetic analysis (Figure 4A, B, C, D; S1B). Our sequence and structure analysis suggest that all REM B3 domains display the family's characteristics fold, as it was observed before for REM 14. Major differences are restricted to the loops where the residues that contact the DNA were identified in RAV1 (Figure 8). The amino acid sequences of REM B3 proteins are poorly conserved and the loops between β strands 1 and 2 and between β strands 4 and 5 and the loop is also shorter in all types of REM B3 domains evaluated by our analysis when compared to RAV1. Although, there are few exceptions, such as the REM IX B and IX C B3 domains of REM37 (At4g31630) which present longer loops (data not shown).

## Discussion

### Evolution of plant B3 superfamily and the first scenario

**Figure 9:**
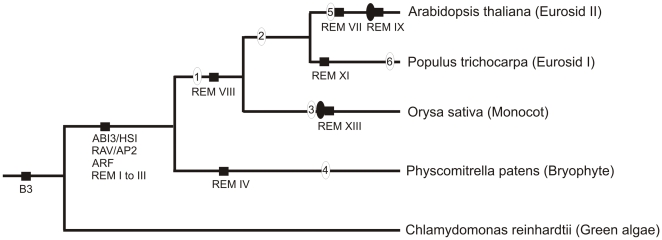
Schematic representation of the phylogeny of the B3 superfamily during plant evolution. Circles with a number inside denote large-scale duplication events: 1, ∼143–220 MYA [52]; 2, ∼66–109 MYA [50], [51]; 3, ∼50–60 MYA [39], [50]; 4, ∼30–60 MYA [53], ∼24–40 MYA [38]; and 6, ∼8–13 MYA [40]. Solid black circles denote recent tandem duplication for B3 genes discovered in this study. The solid black squares, family or class names denote origin of the B3 families during evolution.

We found 87 B3 genes in Arabidopsis (eudicot-eurosids II), 88 B3 genes in poplar (eudicot-eurosids I) and 86 B3 genes in rice (monocots) (Figure 1). We also found 38 genes for the bryophyte *P. patens* and only one gene in the multicellular chlorophyte alga *V. carteri* and the unicellular photosynthetic algae *C. reinhardtii* [35], [42]. The phylogeny of the B3 proteins of all species included in this work indicated that the putative ancestor of the B3 domain is similar to B3 domain of green algae and underwent duplication and evolved to the ABI3 and HSI gene families in plants. As expected, B3 proteins of *C. reinhardtii* and *V. carteri* have a high sequence similarity since both species belong to the Chlorophyta group [12], [43]. In a recent work, Swaminathan and collaborators [30] suggested two possible models of B3 superfamily phylogeny: First, a monophyletic LAV family (LEAFY COTYLEDON2/ABSCISIC ACID INSENSITIVE3 and HSI/VAL) and a sister group relationship between the two algal genes and the VAL group; the second model proposed was based on a tree rooted on the green algal gene. In the second model, a single B3 gene similar in structure to the existing VAL subgroup. Our analysis showed that both are not well supported by the available data. As was mentioned in the previous work [30], it is unlikely that a massive gene loss explains the presence of only one gene in algae giving support to the first model [30] as the higher number of gene family have been increased from algae to flowering plants [25]. The second model is based on the similarity of algae B3 to VAL group of genes, but our sequence comparison and exon/intron analysis and distance calculation (data not shown) strongly suggest that the ancestor of B3 gene from algae underwent a duplication event and gave rise to the ABI3 and HSI families (Figure 7A and 7B). Moreover, the alignment among Arabidopsis, poplar, rice, *P. patens*, *V. carteri* and *C. reinhardtii* showed that the two green algae genes are more similar to ABI3 and HSI groups than any member of the VAL group. Moreover, the exon/intron structure analysis showed that the algae genes have conserved the splicing site position in the first intron when compared to the ABI3 gene family. Despite the conservation in the gene structure and the higher sequence similarity between algae genes and genes of the ABI3 group, our phylogenetic analysis with ABI3 and HSI groups and algae proteins showed a higher similarity between ABI3 and HSI groups than to the algae genes. This indicates that the ABI3 and HSI groups are consequence of an ancient duplication event of an ancestor of the B3 genes which was similar to the algae genes (Figure 9).

By comparing sequences and structural models, it has been suggested that the B3 domain is functionally and structurally similar to the *Eco*RII DNA binding domain [37]. However, until now there is no other evidence to support *Eco*RII gene as the ancestral B3 domain. Perhaps, the horizontal transfer of an ancestral endonuclease *Eco*RII gene into a lower plant may have led to the origin of B3 domain, as has been proposed to explain the origin of other plant transcriptional factor such as WRKY and AP2/ERF [44]. The ancestral B3 domain might have been duplicated to originate the B3 domains of ABI3 and HSI families in bryophytes. A duplication followed by divergence may explain the presence of several distinct characteristics common to ABI3 and HSI families, such as gene structure, protein sequences and the presence of additional domains. Changes in splicing sites are also observed in Arabidopsis HSI genes when compared to other species' HSI genes. As mentioned before, this event might be a result of lineage-specific IS [41]. The IS hypotheses states that new introns positions can arise by relocation of pre-existing introns previously employed as an alternative splicing site. This hypotheses gains influence after the discovery of lineage-specific introns [41], [45]. Interestingly, the presence of alternative splicing in a member of ABI3 family has been recently reported [46].

Our protein modelling analysis of the B3 domains suggest that the B3 domain, including all B3 families and all REM classes, contain virtually identical tertiary structures (the differences are mainly confined to the loops between β strands 1 and 2 and strands 4 and 5, which are thought to interact with DNA [37]) (Figure 8). The observation that some members of the REM family lack conserved amino acids in these two loops has prompted the suggestion that such B3 domains cannot bind DNA. However, in the case of VRN1/REM5, its B3 domain lacks these putative DNA-binding residues and is still capable of binding DNA [13]. Our structural model for the B3 domain of VRN1/REM5 suggests that the domain's characteristic fold is maintained, despite the putative DNA-binding loops being greatly reduced. Taken together, these findings suggest that the B3 domain's characteristic fold may comprise the basic pre-requisites to associate with DNA, while the loops might confer sequence specificity.

The origin of the ARF genes occurred after the appearance of the ABI3 family and must at least predate the origin of mosses. The phylogenetic tree of the ARF family, between Arabidopsis and moss, showed three classes of proteins (I, II and III), suggesting that at least three distinct ARF proteins appeared early in evolution and diverged, resulting in a relatively high number of ARF genes in moss (12 members). In addition, our analysis (phylogenetic and synonymous substitution) suggests that duplication events in ARF genes happened in moss and in Arabidopsis after the divergence between these two species, indicating a smaller number of ARF genes in early periods of evolution of both species. On the other hand, class III from *P. patens* has two ARF genes, which belong to a sister group of *AtARF 10* and *AtARF 16* from Arabidopsis with no duplication events, indicating that in this class the divergence process might have been prevented by functional constraints (See Figure S3). An important question remains about how the B3 domain, the ARF domain, and the III/IV motifs of Aux/IAA appeared in the same gene during evolution. Moss ARF genes have these domains together already. Additionally, Aux/IAA proteins are found in moss [25], indicating an integration of these domains before moss. Nevertheless, we have found three ARF family genes without the ARF domain and III and IV motifs of AUX/IAA in rice (*ARF26*, *ARF27* and *OsARF28*). Synonymous substitutions/site/year analysis strongly suggests that these motifs might have been lost recently.

The complete annotation of *P. patens* genome [25] allowed us to identify members of the RAV family not found before in ESTs libraries [47]. The analysis of the RAV genes' phylogenetic tree (Figure S4) showed two well-supported classes. Interestingly, class I contains all genes with AP2 and B3 domains. The exon-intron structure of Arabidopsis and rice showed that all RAV genes are intronless in the B3 domain. This result suggests that the B3 domain of these genes diverged from the ancient B3 domain, possibly from an ABI3 gene that had lost introns. A phylogenetic analysis of the AP2 domain from all families in *C. reinhardtii* and other derivative species, evidenced a monophyletic origin [48]. These results indicate that the combination of putative intronless B3 and AP2 domains might have first occurred before the emergence of bryophytes.

Analysing the origin and diversification of B3 proteins from REM classes showed that the REM I class is found in moss, rice, poplar and Arabidopsis (Figure 3A). After extensive comparative analysis of REM genes among all species, we also found species-specific REM classes: REM II and IV for moss, REM XII and XIII in rice, REM XI for poplar and REM VII, REM IX and REM X for Arabidopsis. In general, the study of exons/introns from the REM family in highly divergent species such as Arabidopsis, rice and moss have showed a very similar exon-intron structure of the B3 domain, and that the splice site was conserved during plant evolution. Among the REM genes, only *VRN1*, a class VI gene, is well characterized. VRN1 is a protein involved in vernalization-mediated epigenetic silencing of *FLC* [13], [24], [49]. Although there is no functional information about the involvement of other REM gene homologues of *P. patens* and poplar in epigenetic mechanisms, the gene structure conservation between poplar and Arabidopsis for REM VI suggests that this gene may have a function conserved throughout eudicot groups of plants.

Among the REM genes, only three exceptions to the exon-intron structure were observed in Arabidopsis: REM12 and REM 27 are intronless and the genes AtREM1, AtREM2 and AtREM3 have the B3 domain spliced in three exons. REM12 and REM27 are a unique case of loss of the intron in the REM family. REM12 does not have poly-A sequences at the 3′region and also has two introns, which indicates that the intron loss is not a fortuitous event of retroposition (spliced mRNA reversed-transcribed and inserted into a new genomic position). It has been suggest that the loss of an individual intron may be the result of a nonhomologous recombination stimulated by the common occurrence of short direct repeats in or near the 5′ and 3′ splice sites [41]. AtREM1, AtREM2 and AtREM3 have the B3 domain spliced in three exons. Their splice site are conserved when compared with other B3 genes (Figure 4A and E). Moreover, the phylogenetic analysis grouped them together with rice genes that also possess the same gene structure, the B3 domain spliced in three exons (OsREM1, OsREM2, OsREM3, OsREM4) (Figure 4E), indicating that these two groups of genes share a common B3 ancestral gene with more splice sites. The presence of a higher number of REM genes with a reduced number of introns is in agreement with results that indicate that intron losses have outnumbered intron gains in several gene families during plant evolution [41], [45].

### Timing duplication and DNA binding of plant B3 domain

We investigated the duplication events in Arabidopsis, poplar, rice and moss by using calculated synonymous substitutions/site/year (Table S6, see Figure 5). We observed three distinct patterns of duplication events for B3 genes according to their duplication origin: genes that were duplicated and inserted in different chromosomes, genes that were duplicated and inserted in the same chromosome and genes that were duplicated in tandem. The phylogenetic tree of B3 proteins for each family shows that many genes of the ABI3, HSI, ARF, RAV and REM families have underwent duplication events after the split between bryophytes and angiosperms, monocots and eurosids, and eurosids I and eurosids II, showing that the expansion of the B3 superfamily occurred in several stages (Figure 9).

The B3 phylogeny is consistent with other studies of plant evolution, which suggest that this family of genes might be associated with development of innovative function in plants [38], [39], [40], [50], [51], [52], [53]. The ABI3 ancestral gene originated the ARF, RAV, REM genes. It is interesting to note that several distinct B3 domains and the association with another domain are already found in moss. This elevated diversity may be associated with the transition of aquatic green algae to terrestrial plants. After the split between bryophyte and angiosperms, the first REM class to emerge in angiosperms, approximately 134–220 MYA, was REM VIII. Other species-specific classes, such as REM VII and REM IX in Arabidopsis, REM XI in poplar, REM XIII in rice and REM IV in moss appeared later in evolution (Figure 9). Based on phylogenetic trees and calculate synonymous substitutions/site/year, we propose that REM IX from Arabidopsis and REM XIII from rice suffered later genome duplication events originating a new species-specific REM class. Two other species-specific classes, REM VII of Arabidopsis and REM XI of poplar, have probably emerged from other processes, such as tandem duplications (see above).

As described above, the REM family results from a very dynamic evolution, involving many genes with several of them displaying more than one B3 domain per protein. Our analysis showed that the B3 domain from REM I, REM-A type, is present in moss, rice, poplar and Arabidopsis. The phylogenetic tree with all of the B3 domains of *P. patens* did not reveal the ancestral REM B3 domain, although it is clear that this family diverged in mosses. The phylogeny of the REM proteins also reveals a very active and dynamic process of gene duplication. This process resulted in the portrait of the REMs in plants, a large number of genes with a remarkable variability among them.

Genome or tandem duplication may explain the emergence of the large number of REM genes, but what causes their maintenance as active genes in the genome is still an open question. It has been suggested that, after evaluation of MADS box genes' phylogenetic and expression analysis, subfunctionalization and/or neofunctionalization play a role in the maintenance of most of the duplicated regulatory genes in Arabidopsis [54]. On the other hand, Wellmer and collaborators [55] suggested that the functional redundancy during early flower development may have increased the genetic buffering so that duplicated genes are retained by positive selection. They identified, by global analysis of gene expression, a significant enrichment of transcription factor families with closely related members expressed in early Arabidopsis flower development [55]. The maintenance REM family gene members may be a combination of the subfunctionalization and/or neofunctionalization as well as the genetic buffering processes. The elucidation of the phylogeny of this complex gene family will greatly assist strategies for the study of the functional importance of REM genes during early flower development.

### Evolution and function of plant B3 proteins

The phylogenetic comparative analysis of *Arabidopsis*, poplar, rice, *P. patens*, *V. carteri* and *C. reinhardtii* genes revealed that the ancestral B3 domain gave rise, after gene duplication, to ABI3 and HSI families. One obvious question is what is the function of B3 proteins in algae? It is known that ABI3 protein is involved with several functions including plastid development in higher plants which may also be important for algae [56], [57]. ABI3 is expressed in several tissues indicating that it might be also involved with other central functions in plant life not yet uncovered. The algae genes identified up to now have a high similarity to ABI3 group member suggesting that the algae B3 might be involved in plastid development or other still unknown crucial functions. Marella collaborators [58] sequenced three similar B3 proteins in *P. patens* belonging to the ABI3 family and showed that *PpABI3A* can partially complement the Arabidopsis *abi3-6* mutant [58]. *ABI3*, *FUS3* and *LEC2* genes, all members of the ABI3 class, are master regulators of the maturation phase during embryogenesis [59]. It has been shown that *FUS3* and *LEC2* are involved in the repression of gibberellin biosynthesis in Arabidopsis [60], [61]. Our AtGenExpression analysis for ABI3 and HSI genes (Table S7) also showed an overlapping expression pattern during seed development for all ABI3 genes and one HSI gene.

Genes from the RAV and REM families are not well studied. NGA genes from the RAV family were described as redundantly regulating lateral organ growth [18]. Additionally, *TEM1* and *TEM2* have been showed as direct repressors of *FT*, participating in a quantitative balance between *CO* (*CONSTANS*) and *TEM* to determine the threshold level required for flowering [19]. On the other hand, members of the REM family have a poorly conserved B3 domain when compared with other B3 families. Variability in conserved domains is usually associated with flexibility in interactions with DNA or in protein-protein interaction [62], [63], which might indicate a functional innovation in the REM family and a diversification of the B3 domain.

Do members of the B3 REM family bind to a specific DNA site? This question is still unanswered. Cobbler consensus sequences of each AtREM class illustrate a high variability in the B3 domain, including the residues in critical positions for DNA interaction. Our protein modelling analysis reveals that all REM B3 domains contain identical tertiary structures and the differences are restricted to the loops that are shorter than the RAV1 protein. Our analysis included VRN1, an AtREM VI class member that has the capacity to generally bind DNA [13]. However, there is no evidence that VRN1 can recognize a specific site in the tested conditions [13]. VRN1, together with VERNALIZATION 2 and LIKE HETEROCHROMATIN PROTEIN 1 (LHP; also known as TFL2) are required for maintenance of *FLC* (*FLOWERING LOCUS C*) silencing [13], [24], [64].

The only other functional information about REM family members is the expression pattern. A member of the REM IX class (REM 34) is expressed in the vegetative apical meristem, later expanding to the whole inflorescence meristem [7], which is the same expression pattern found in AtGenExpress (Table S7). Many REM VIII and REM VII genes are expressed during early Arabidopsis flower development [55], [65], [66]. *In situ* hybridization experiments showed that REM 22, 23, 25, 13, 15 and 16 have unique spatial expression patterns during early stamen and carpel development. *In silico* analysis also indicates that overlapping expression patterns are widely spread in REM family (Table S6). Their partially overlapping *in situ* and *in silico* expression patterns and high sequence similarity indicate functional redundancy during shoot apical meristem, flower and reproductive development. It would be interesting to investigate whether REM VIII and REM VII members are also involved in epigenetic maintenance, as it was demonstrate for *VRN1*. Their expression patterns, restricted to few cells during particular developmental stages, suggest the possibility that they may be involved with epigenetic regulation of gene expression during cell differentiation.

## Materials and Methods

### Gene and domain identification

Arabidopsis proteins containing B3 domains were obtained from the Arabidopsis Information Resources (TAIR; http://www.arabidopsis.org/tools/bulk/protein/index.jsp) using the INTERPRO code IPR003340 for B3 domain (http://www.ebi.ac.uk/interpro/). The exon/intro structures were investigated using SeqViewer at TAIR (http://www.arabidopsis.org/servlets/sv) and the Munich Information Center for protein sequence (MIPS) (http://mips.gsf.de/proj/plant/jsf/rice/searchjsp/index.jsp) for comparative analysis.

For the red alga *Cyanidioschyzon merolae,* we conducted a BLASTP using different B3 domains of *Arabidopsis* (http://merolae.biol.s.u-tokyo.ac.jp/blast/blast.html). For the green algae *Chlamydomonas reinhardtii*, *Volvox carteri*, *Ostreococcus tauri* and *Ostreococcus lucimarinus*, B3 genes were obtained using the Interpro code IPR003340 for B3 domain (http://genome.jgi-psf.org/Chlre3/Chlre3.home.html). To further confirm our search results, we conducted a TBLASTN search using different B3 domains of Arabidopsis. For the moss *Physcomitrella patens*, B3 genes were obtained using the Interpro code IPR003340 for B3 domain (http://genome.jgi-psf.org/Phypa1_1/). We also confirmed our results by searching with selected sequences the Physcomitrella EST Project Web site (http://moss.nibb.ac.jp/), the NCBI (National Center for Biotechnology Information) and the sputnik EST (http://mips.gsf.de/proj/sputnik/) data banks. For the monocot *Orysa sativa,* proteins containing B3 domains were obtained at the Institute for Genomic Research (TIGR) (http://www.tigr.org/tdb/e2k1/osa1/domain_search.shtml) using the PFAM code PF02362 for B3 domain. The exon/intron structures were investigated using MIPS. *Populus trichocarpa* B3 genes were obtained using the Interpro code IPR003340 for B3 domain (http://genome.jgi-psf.org/Poptr1/Poptr1.home.html). The exon/intron structures of *C. reinhardtii*, *V. Carteri* and *P. patens* were investigated using their databases.

### Annotation of domains, sequence analysis, alignment and construction of phylogenetic trees

The sequence coordinates of B3, ARF and AP2 domains plus E-value showed for B3 domain were annotated according to Pfam databases [67]. B3 genes that contained more than one B3 domain had their B3 domain treated separately as an operational taxonomic units (OTUs). Each B3 domain was identified by numbers according to their position starting form amino terminal). The ARF domain used in our analysis was based on PFAM annotation and contained from 80 to 84 aminoacids. The AUX/IAA III and IV domains were annotated according to the alignment of our sequences and sequences described in Ulmasov *et al*. [11]. Multiple alignments with complete sequences or domains were conducted using the CLUSTALW program [68] using default parameters and then manually revised. Phylogenetic trees were constructed using the neighbor-joining method [31] and *p*-distance on the Mega 4.1 program [32]. Assessment of node confidence was done by means of 1,000 bootstrap replicates. In order to verify topological stability, we have also conducted phylogenetic analyses using the JTT model [33], which was chosen by the Akaike information criterion on the ProtTest software [69]. All topologies inferred were robust to model assumption. To investigate the physiochemical amino acid properties, we conducted the analysis of B3 domain consensus sequences in GeneDoc [70]. The consensus of each family/class was generated by the COBBLER program using all the B3 domain sequence data available for each family/class [36].

### Timing of gene duplication events

To investigate the age of the duplication events between paralogous copies of the newly discovered B3 genes, we adopted the approach of Blanc and Wolfe (2004) [50]. If the synonymous distance accumulates approximately linearly with time, it can be used to infer divergence times using the equation *d*_S_ = 2 µ_S_ *T*, with the mean rate of synonymous evolution set at 1.5×10^−8^ substitutions/synonymous site/year for eudicots [71] and 6.5×10^−9^ substitutions/synonymous site/year for monocots [72]. This mathematical equivalence should hold when the estimated *d*_S_ is not greater than 2, which indicates that the estimate distance is not saturated. Although error prone, this strategy may offer an approximate evolutionary scenario of B3 domain and gene evolution. Paralogous gene copies of B3 genes were obtained by blasting each genome against itself to identify best bidirectional hits [73]. We downloaded EST data sets of *Arabidopsis* from TAIR (TAIR6_cds_20051108), rice ones from TIGR (all.cds), and *Populus* (transcripts.Poptr1_1.JamboreeModels.fasta) and *Physcomitrella* (transcripts.Phypa1_1.FilteredModels.fasta) from JGI.

To determine the location of the B3 domain in five chromosomes of *Arabidopsis*, we used the Chromosome Map Tool (http://www.arabidopsis.org/jsp/ChromosomeMap/tool.jsp). Gene duplication and their presence on duplicated segments were investigated using the MIPS Interactive Redundancy Viewer (http://mips.gsf.de/proj/thal/db/gv/rv/rv_frame.html) and the “Paralogous in Arabidopsis” (http://wolfe.gen.tcd.ie/athal/dup) as defined by Blanc et al. [38].

### *In silico* analysis of expression pattern

The expression mean-normalized values was download from AtGenExpress Visualization Tool (AVT) [74] for all B3 present in these databank. All significative tissue and specific and transition developmental stage was considered in these analysis to verify where ABI3, HSI, ARF, RAV and REM genes are presents. The colour used to highlight the expression data was used using three criteria. We have classified gene expression patterns in three categories, namely, low, medium and high expression. For each gene we firstly identified the maximum expression value recorded. The low category includes genes with expression patterns between 0 and a third of the maximum value; medium category genes presented expression patterns greater than a third of the maximum and lower than two-thirds of the maximum. Finally, if the value was greater than two-thirds of the maximum, it was classified as highly expressed.

### Strutural Modeling of B3 domains

Domains B3 from selected members of the REM family of plant proteins were defined as target sequences (see text for accession numbers). The NMR solution structures for the Arabidopsis REM protein At1g16640 (residues 1 to 102; PDB ID 1YEL) or RAV1 (residues 182 to 295; PDB ID 1WID) were used as template [12], [37]. EXPRESSO (3DCoffee) [75] was used to align the B3 domain of At1g16640 (residues 1 to 102) to the equivalent domain from various members of the REM protein family. The quality of the target-template alignment was further assessed by comparing the structurally determined (STRIDE) [76] and predicted (PSIPRED) [77], [78], [79] secondary structures. The target-template alignment was used to build the model in Swiss Model [80], [81]. Local model quality was estimated using ProqRes [82] and ANOLEA [83]. QMEAN [80] and DFire [84] were used to estimate global model quality. Finally, Procheck [85] was used to assess the conformational quality of the models. Structural alignments were performed with SSM [86]. All figures depicting structural models were prepared using Pymol (http://www.pymol.org/).

## Supporting Information

Figure S1 Phylogenetic relationships of B3 proteins and B3 domain. A, Unrooted Neighbor-joining tree of the entire amino acid sequences of P. patens REM family showing four different classes well supported. PpREM12 is long and unique protein, for this reason it is not included to anyone of the typical classes. It encloses three B3 domains, two of them are similar and grouped in REM II B type and the N-terminal B3 domain belongs to REM III A type (Figure 3A). B, Unrooted Neighbor-joining tree of the entire amino acid sequences of A. thaliana REM family showing five different classes well supported. The other REM V and REM X were excluded from this analysis because they have some differences of amino acids that disturbe the alignment and tree topology. REM V and REM X clusteres in specific branch (data not shown). C, Rooted in ABI3/HSI Neighbor-joining tree of all seven B3 families based on the whole B3 domain amino acid sequences of A. thaliana. D, Rooted in ABI3/HSI Neighbor-joining tree of all five B3 families based on the whole B3 domain amino acid sequences of rice. Bootstrap values from 1,000 replicates were used to assess the robustness of the trees. Bootstrap values >40 are shown. The scale bar represents a 0.1 estimated amino acid substitution per residue.
(2.86 MB TIF)

Figure S2 Phylogenetic relationships among Arabidopsis and rice B3 protein sequences from group ABI3/HSI, ARF and RAV. A, Unrooted Neighbor-joining tree of the entire amino acid sequences of Arabidopsis ABI3/HSI proteins. B, Unrooted Neighbor-joining tree of the entire amino acid sequences of Arabidopsis ARF proteins. C, Unrooted Neighbor-joining tree of the entire amino acid sequences of Arabidopsis RAV proteins. D, Unrooted Neighbor-joining tree of the entire amino acid sequences of rice ABI3/HSI proteins. E, Unrooted Neighbor-joining tree of the entire amino acid sequences of rice ARF proteins. F, Unrooted Neighbor-joining tree of the entire amino acid sequences of rice RAV proteins. Bootstrap values from 1,000 replicates were used to assess the robustness of the trees. Bootstrap values >50 are shown. The scale bar represents a 0.1 estimated amino acid substitution per residue. The phylogenetic tree and exon/intron structure with domain localization of every group, ABI3, HSI, ARF, RAV are shown. Each colored box represent B3, ARF, AP2, Aux/IAA III and IV domains as indicate in the figure. MIPS Arabidopsis thaliana and Orysa sativa database was used for exon/intron structure information [87].
(3.21 MB TIF)

Figure S3 Phylogenetic relationships among Arabidopsis and P. patens B3 protein sequences from the ARF family. Unrooted Neighbor-joining tree of the entire amino acid sequences of ARF proteins. Bootstrap values from 1,000 replicates were used to assess the robustness of the trees. Bootstrap values >50 are shown. The scale bar represents a 0.05 estimated amino acid substitution per residue. Classes are denoted by roman algorisms I, II and III, and sub-classes by capital letters A to D.
(0.95 MB TIF)

Figure S4 Phylogenetic relationships among Arabidopsis, rice, poplar and P. patens B3 protein sequences from the RAV family. Unrooted Neighbor-joining tree of the entire amino acid sequences of RAV proteins. Bootstrap values from 1,000 replicates were used to assess the robustness of the trees. Bootstrap values >50 are shown. The scale bar represents a 0.05 estimated amino acid substitution per residue. Classes are denoted by roman algorisms I and II.
(1.05 MB TIF)

Table S1 Physcomitrella patens B3 gene list. Gene ID and additional information about the P. patens B3 genes used in this study. The source for all sequences was the JGI data bank. Marella and collaborators [58] isolated three complete sequences for B3 proteins (AB233419, AB233420 and AB245516) that belong to the ABI3 class [58]. Our comparative analysis showed that AB233419 is ABI3 A. AB233420 (ABI3 B) is similar to [Phypa1_1:168363] with one amino acid changed in position 55, from W to R, respectively. AB245516 (ABI3 C) is similar to [Phypa1_1:86215] with over 19 amino acids at position 55 for [Phypa1_1:86215].
(0.04 MB XLS)

Table S2 Arabidopsis thaliana B3 gene list. Gene ID and additional information about the A. thaliana B3 genes used in this study. The source for all sequences was the TAIR data bank.
(0.05 MB XLS)

Table S3 Populus trichocarpa B3 gene list. Gene ID and additional information about poplar B3 genes used in this study. The source for all sequences was the JGI data bank.
(0.05 MB XLS)

Table S4 Orysa sativa B3 gene list. Gene ID and additional information about rice B3 genes used in this study. The source for all sequences was the TIGR data bank. Asterisks denote protein not found by Swaminathan et al. [30].
(0.05 MB XLS)

Table S5 List of B3 genes found by additional resources and putative domain found. List of the additional B3 genes found by Swaminathan et al. [30] for Arabidopsis and rice. Identification of domain and E-value was conducted in Pfam analysis. Genes with putative B3 domains or other DUF domain with not significant E-value were not included in our analysis. Asteristics represents pseudogenes described by TAIR.
(0.03 MB XLS)

Table S6 List of detected age estimation in B3 proteins. dS, YA and MYA means synonimous distance, years and million years ago.
(0.10 MB XLS)

Table S7 In silico Expression analysis of B3 genes. Gene ID and additional information about the A. thaliana B3 genes used in this study. The source for all sequences and mean-value expression was the TAIR data bank and AtGenExpress [74], respectively.
(0.04 MB XLS)
