# Supplementary figures and images for: Evolution of the B3 DNA Binding Superfamily: New Insights into REM Family Gene Diversification
Source: PLoS One. 2009 Jun 8;4(6):e5791. doi: 10.1371/journal.pone.0005791 (PMC2688026; doi:10.1371/journal.pone.0005791)

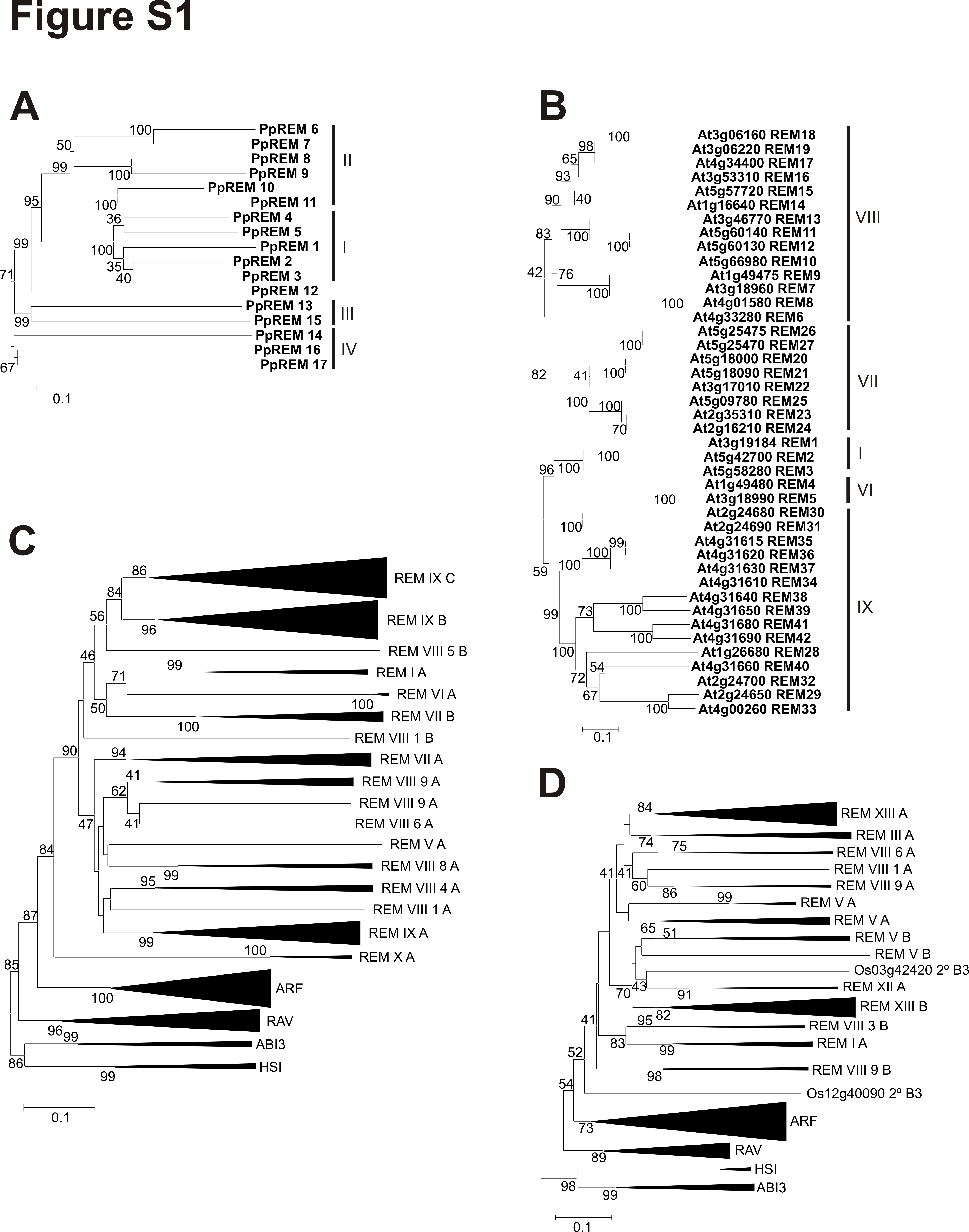

Supplement: Figure S1 — Phylogenetic relationships of B3 proteins and B3 domain. A, Unrooted Neighbor-joining tree of the entire amino acid sequences of P. patens REM family showing four different classes well supported. PpREM12 is long and unique protein, for this reason it is not included to anyone of the typical classes. It encloses three B3 domains, two of them are similar and grouped in REM II B type and the N-terminal B3 domain belongs to REM III A type (Figure 3A). B, Unrooted Neighbor-joining tree of the entire amino acid sequences of A. thaliana REM family showing five different classes well supported. The other REM V and REM X were excluded from this analysis because they have some differences of amino acids that disturbe the alignment and tree topology. REM V and REM X clusteres in specific branch (data not shown). C, Rooted in ABI3/HSI Neighbor-joining tree of all seven B3 families based on the whole B3 domain amino acid sequences of A. thaliana. D, Rooted in ABI3/HSI Neighbor-joining tree of all five B3 families based on the whole B3 domain amino acid sequences of rice. Bootstrap values from 1,000 replicates were used to assess the robustness of the trees. Bootstrap values >40 are shown. The scale bar represents a 0.1 estimated amino acid substitution per residue. (2.86 MB TIF) [file pone.0005791.s001.tif]

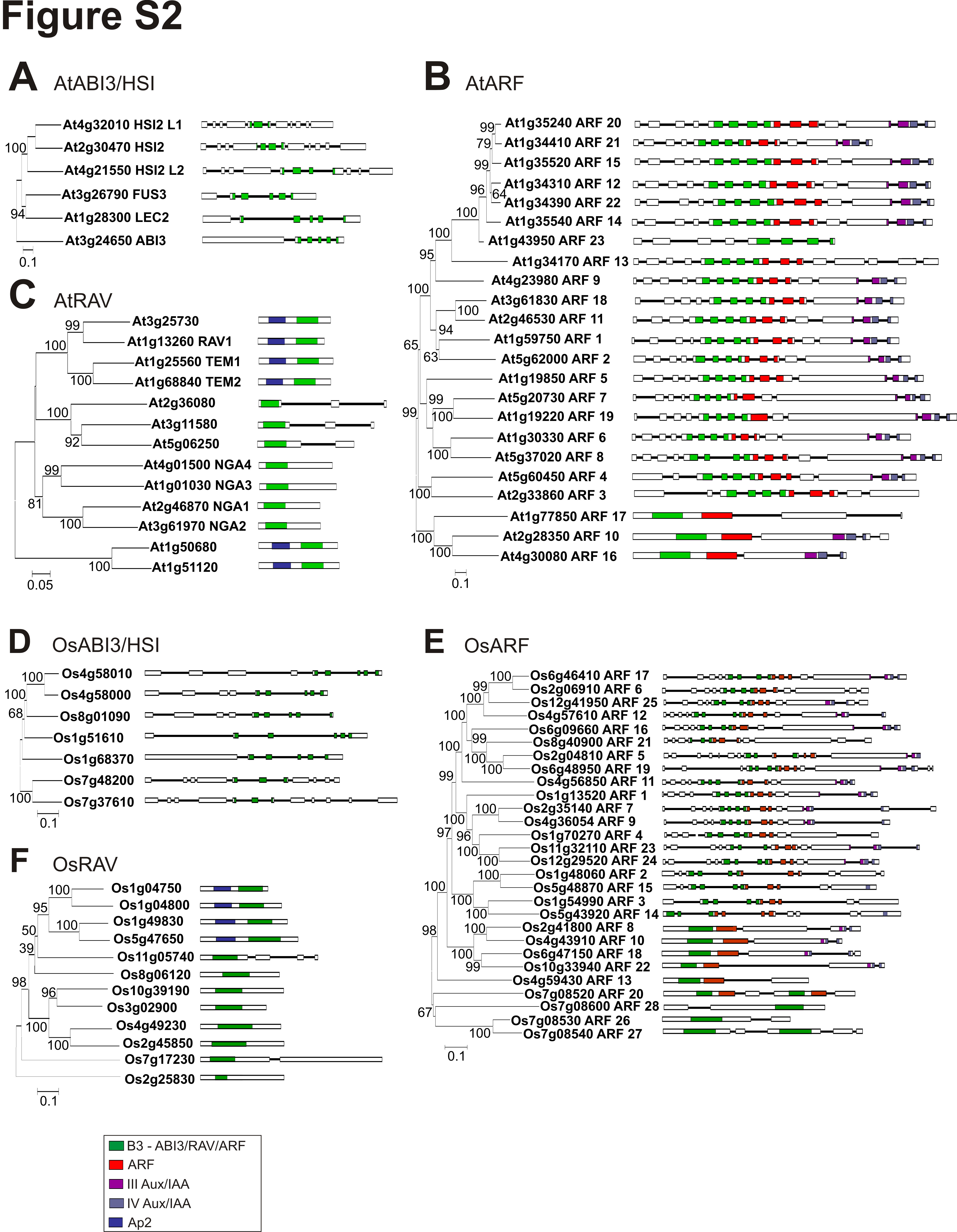

Supplement: Figure S2 — Phylogenetic relationships among Arabidopsis and rice B3 protein sequences from group ABI3/HSI, ARF and RAV. A, Unrooted Neighbor-joining tree of the entire amino acid sequences of Arabidopsis ABI3/HSI proteins. B, Unrooted Neighbor-joining tree of the entire amino acid sequences of Arabidopsis ARF proteins. C, Unrooted Neighbor-joining tree of the entire amino acid sequences of Arabidopsis RAV proteins. D, Unrooted Neighbor-joining tree of the entire amino acid sequences of rice ABI3/HSI proteins. E, Unrooted Neighbor-joining tree of the entire amino acid sequences of rice ARF proteins. F, Unrooted Neighbor-joining tree of the entire amino acid sequences of rice RAV proteins. Bootstrap values from 1,000 replicates were used to assess the robustness of the trees. Bootstrap values >50 are shown. The scale bar represents a 0.1 estimated amino acid substitution per residue. The phylogenetic tree and exon/intron structure with domain localization of every group, ABI3, HSI, ARF, RAV are shown. Each colored box represent B3, ARF, AP2, Aux/IAA III and IV domains as indicate in the figure. MIPS Arabidopsis thaliana and Orysa sativa database was used for exon/intron structure information [87]. (3.21 MB TIF) [file pone.0005791.s002.tif]

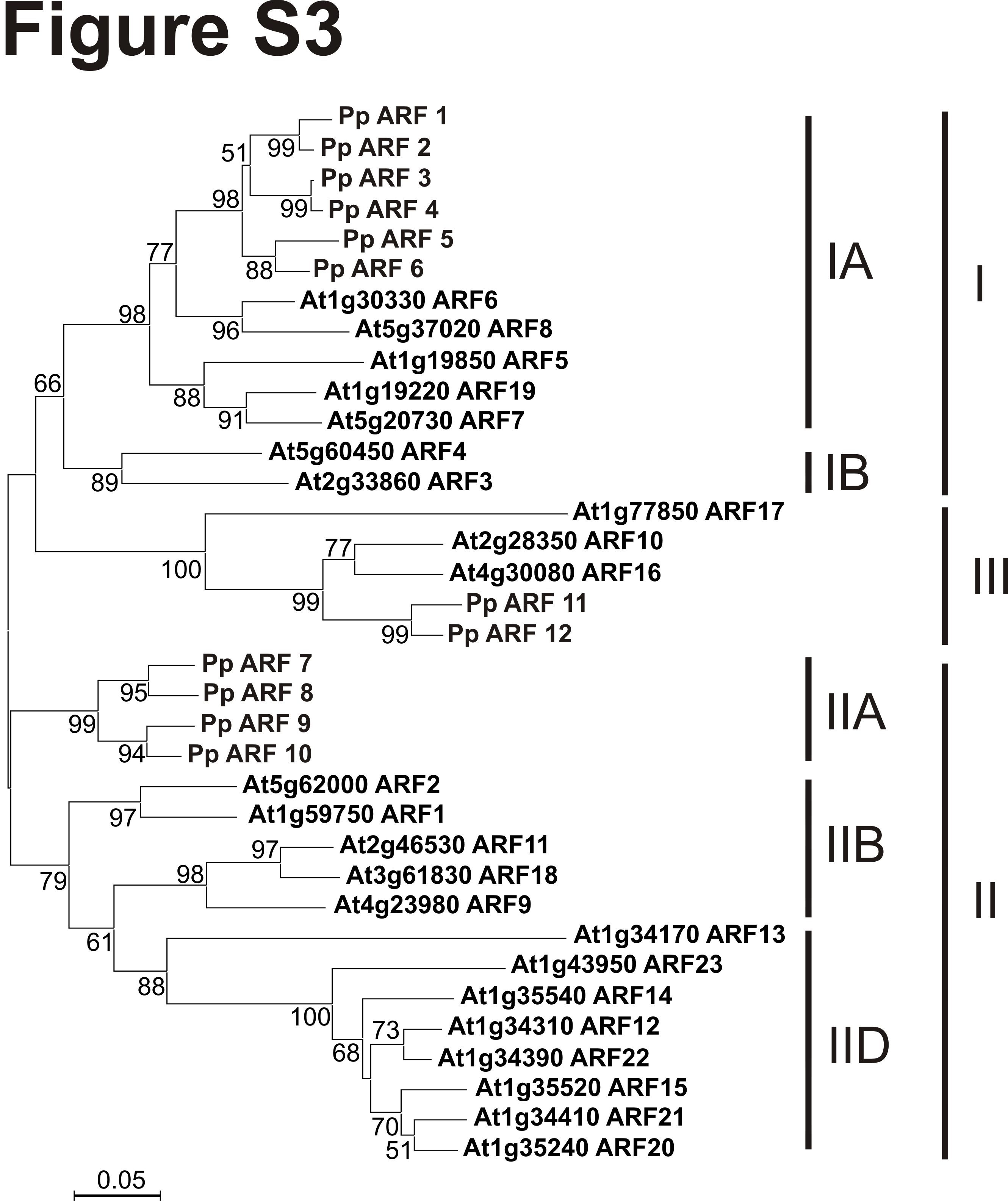

Supplement: Figure S3 — Phylogenetic relationships among Arabidopsis and P. patens B3 protein sequences from the ARF family. Unrooted Neighbor-joining tree of the entire amino acid sequences of ARF proteins. Bootstrap values from 1,000 replicates were used to assess the robustness of the trees. Bootstrap values >50 are shown. The scale bar represents a 0.05 estimated amino acid substitution per residue. Classes are denoted by roman algorisms I, II and III, and sub-classes by capital letters A to D. (0.95 MB TIF) [file pone.0005791.s003.tif]

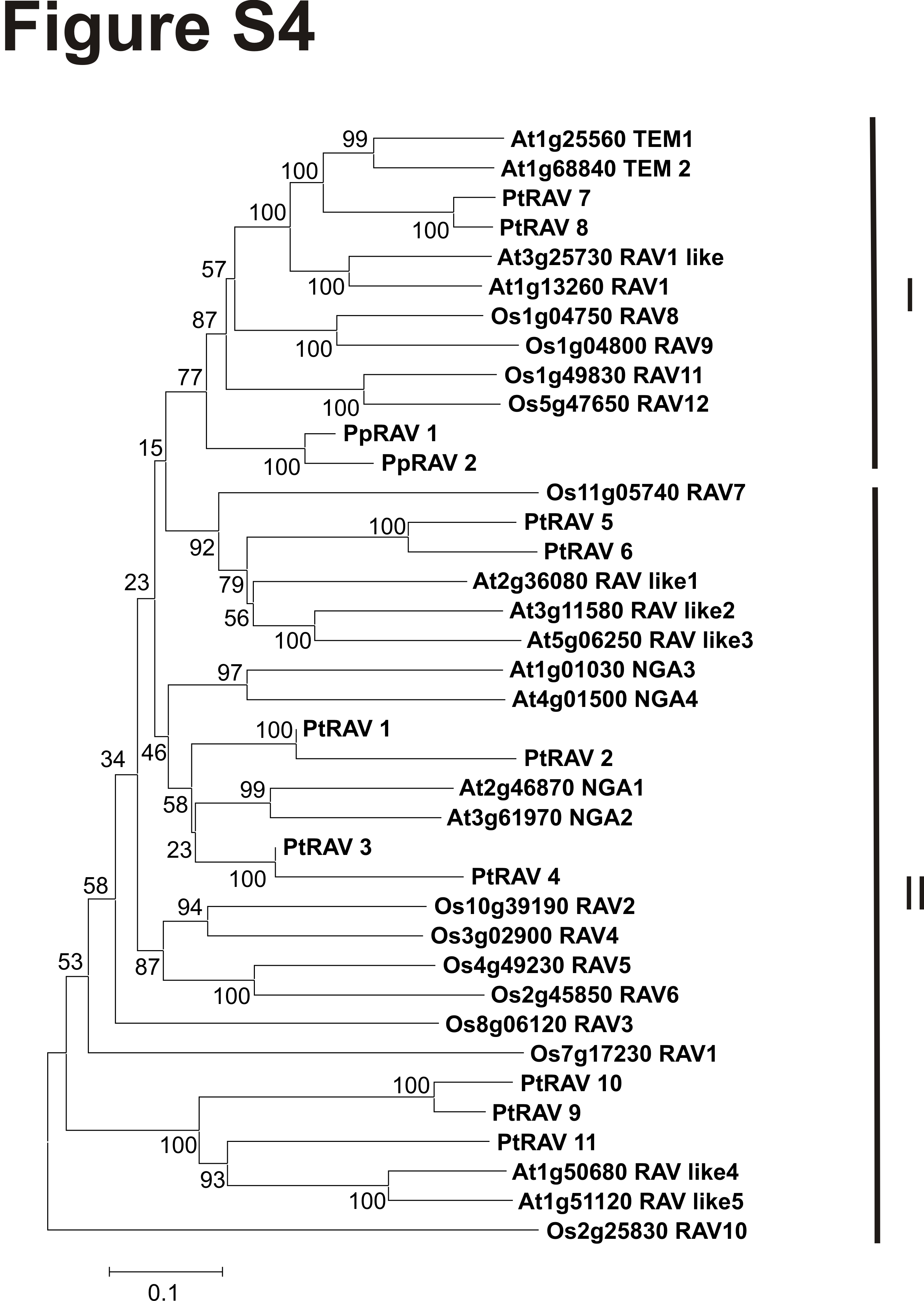

Supplement: Figure S4 — Phylogenetic relationships among Arabidopsis, rice, poplar and P. patens B3 protein sequences from the RAV family. Unrooted Neighbor-joining tree of the entire amino acid sequences of RAV proteins. Bootstrap values from 1,000 replicates were used to assess the robustness of the trees. Bootstrap values >50 are shown. The scale bar represents a 0.05 estimated amino acid substitution per residue. Classes are denoted by roman algorisms I and II. (1.05 MB TIF) [file pone.0005791.s004.tif]
